# Supplementary material for: Homopolymer switches mediate adaptive mutability in mismatch repair-deficient colorectal cancer
Source: Nat Genet. 2024 Jul 3;56(7):1420–33. doi: 10.1038/s41588-024-01777-9 (PMC11250277; doi:10.1038/s41588-024-01777-9)
Supplement: Supplementary file 1 — Supplementary Notes 1–4. [file 41588_2024_1777_MOESM1_ESM.pdf]

nature genetics

Supplementary information

https://doi.org/10.1038/s41588-024-01777-9

Homopolymer switches mediate adaptive mutability in mismatch repair-deficient colorectal cancer

In the format provided by the authors and unedited

Supplementary Note

This supplementary note has additional information on:

1. **Analysis of cases with confirmed MutLα loss in GEL WGS cohort**
2. ***In vivo* estimation of intra-tumour mutation rate using MOBSTER**
3. **Immune dNdS analysis**
4. **Mathematical model of mutation rate switching**

1. Analysis of cases with confirmed MutL$_\alpha$ loss in GEL WGS cohort

Stratifying by *MSH3* and/or *MSH6* homopolymer frameshift mutation status in the Genomics England CRC WGS cohort (n=217) demonstrated a clear stepwise increase for SNVs, indels, and overall mutation burden with incremental MMR homopolymer mutations (**Fig. 1F-H**). As a further control, we wanted to restrict our analysis to cases with confirmed truncal loss of MutL$_\alpha$ (MLH1/PMS2). In the Genomics England CRC WGS cohort, we lacked epigenetic data on *MLH1* methylation status and therefore used somatic *BRAF*^{V600E} mutation as a proxy for *MLH1* methylation in MSI tumours (Adar et al, *Mod. Pathol.* **30,** 440–447, 2017, and Salem, M. E. *et al. Int. J. Cancer* **30,** v403–9, 2020). Reassuringly, analysing cases with either *BRAF*^{V600E} mutation or germline *MLH1* or *PMS2* pathogenic mutations (total n=135) corroborated the relationship between *MSH6*^{F1088fs} / *MSH3*^{K383fs} and increased mutation burden (**Extended Data Fig. 1A-C**).

2. *In vivo* estimation of intra-tumour mutation rate using MOBSTER

We wanted to derive clone-specific *in vivo* mutation rates comparing MSH6-proficient and MSH6-deficient regions in individual tumours. In order to retrieve *in vivo* mutation rate estimates from single timepoint sequencing data, we developed a pipeline around MOBSTER, a recently published computational method that performs tumour subclonal deconvolution by integrating population genetics and machine learning (Caravagna, G. *et al. Nature Genetics* **52,** 898–907, 2020). This method retrieves a mutation rate estimate from the tail of neutral mutations within the allele frequency spectrum of a given sample. Specifically, we applied a population genetics approach to estimate sample-specific mutation rates μ from the fit of neutral tails, which we normalised for the portion of the diploid genome that had undergone WXS sequencing (see Methods for details). Comparison of mutation rate values between samples of patient UCL-1014 (**Fig. 3L**) shows that μ = 2.11^{-7} for the MSH6-proficient sample, whilst μ =1.51^{-6} for the MH6-deficient sample (**Fig. 3M-O**). The confidence intervals (bootstrapped percentile from 200 resamples, see Methods) for both estimates were non-overlapping with a difference of over one order of magnitude between μ point estimates (**Fig. 3P**). These results provide *in vivo* evidence that MMRd tumours are a mosaic of varying mutation rates and mutation biases. This heterogeneity is masked in bulk tumour analyses.

3. Immune dNdS analysis

We sought to test whether immune selection favours incremental MMR homopolymer indels by using immune dNdS analysis. Immune dNdS measures the ratio of

nonsynonymous to synonymous mutations at genomic loci that are exposed to the immune system (Immune ON) compared to neutral expectation (Zapata, L. *et al. Nature Genetics* **55,** 451–460, 2023). Here we calculated dNdS across genomic regions that bind to HLA-A0201, the most common HLA class I allele in the Caucasian population, to compare patients across our cohort. As an internal control, Immune ON dNdS is compared to dNdS values in genomic coding regions outside of those exposed to the immune system (Immune OFF) (**Extended Data table 7**). We first examined tumour regions without *MSH6*^{F1088fs} or *MSH3*^{K383fs} and found no significant difference in nonsynonymous mutation accumulation between genomic regions exposed to the immune system (Immune ON) and those not exposed to the immune system (Immune OFF) (**Fig. 5P**). By contrast, regions with *MSH6*^{F1088fs} and/or *MSH3*^{K383fs} showed a highly significant difference when comparing Immune ON and Immune OFF dNdS (**Fig. 5P**). These molecular evolution data thus show enrichment of nonsynonymous mutations in HLA-A0201-binding regions in subclones with *MSH6*^{F1088fs} and/or *MSH3*^{K383fs}. This supports subclonal selection of these MMR homopolymer indel mutations through linked immune escape variants. Overall, these data indicate that evolving mismatch repair-deficient cancers elicit immune escape by balancing the evolutionary costs of adaptive hypermutability (increased deleterious genomic mutation load and immune cell infiltration) against its fitness advantages (greater subclonal HLA and neoantigen diversity).

4. Mathematical model of mutation rate switching

To explore the impact of mutation rate switching on tumour evolution, we examined tumour growth dynamics as extension of our previously published model (Lakatos, E. *et al. Nature Genetics* **52,** 1057–1066, 2020). We traced the number of lineages that were eliminated until 10 lineages had survived and repeated this analysis along a range of switch rates. As cells experiencing higher mutation rate regimes have a higher probability of gaining an immune escape mutation, we hypothesised and confirmed that stochastic switching to a higher mutation rate decreases the rate of immunogenic lineage extinction (**Extended Data Fig. 8A**). Switching did not affect tumours with a starting population experiencing the higher mutation rate regime (**Extended Data Fig. 8B**), as these tumours carried a significant proportion of fast mutating cells regardless of switching rate. In tumours with a lower initial mutation rate, on the other hand, frequent switching leads to only small (although still significant) changes in growth time (**Extended Data Fig. 8C**).
