# Supplementary material for: Homopolymer switches mediate adaptive mutability in mismatch repair-deficient colorectal cancer
Source: Nat Genet. 2024 Jul 3;56(7):1420–33. doi: 10.1038/s41588-024-01777-9 (PMC11250277; doi:10.1038/s41588-024-01777-9)
Supplement: Supplementary file 2 — Reporting Summary [file 41588_2024_1777_MOESM2_ESM.pdf]

Corresponding author(s): Marnix Jansen, Hugo Snippert

Last updated by author(s): Jan 27, 2024

Reporting Summary

Nature Portfolio wishes to improve the reproducibility of the work that we publish. This form provides structure for consistency and transparency in reporting. For further information on Nature Portfolio policies, see our Editorial Policies and the Editorial Policy Checklist.

Statistics

For all statistical analyses, confirm that the following items are present in the figure legend, table legend, main text, or Methods section.

n/a	Confirmed	
☐	☒	The exact sample size (*n*) for each experimental group/condition, given as a discrete number and unit of measurement
☐	☒	A statement on whether measurements were taken from distinct samples or whether the same sample was measured repeatedly
☐	☒	The statistical test(s) used AND whether they are one- or two-sided<br>*Only common tests should be described solely by name; describe more complex techniques in the Methods section.*
☐	☒	A description of all covariates tested
☐	☒	A description of any assumptions or corrections, such as tests of normality and adjustment for multiple comparisons
☐	☒	A full description of the statistical parameters including central tendency (e.g. means) or other basic estimates (e.g. regression coefficient) AND variation (e.g. standard deviation) or associated estimates of uncertainty (e.g. confidence intervals)
☐	☒	For null hypothesis testing, the test statistic (e.g. *F*, *t*, *r*) with confidence intervals, effect sizes, degrees of freedom and *P* value noted<br>*Give P values as exact values whenever suitable.*
☒	☐	For Bayesian analysis, information on the choice of priors and Markov chain Monte Carlo settings
☒	☐	For hierarchical and complex designs, identification of the appropriate level for tests and full reporting of outcomes
☒	☐	Estimates of effect sizes (e.g. Cohen's *d*, Pearson's *r*), indicating how they were calculated

Our web collection on statistics for biologists contains articles on many of the points above.

Software and code

Policy information about availability of computer code

Data collection	Alignment of FASTQ sequencing files: BWA-mem (version 0.7.7) (http://bio-bwa.sourceforge.net/)<br>Sorting and indexing of BAM files: SAMtools (http://www.htslib.org/)<br>Marking duplicate reads and local INDEL realignment: PICARD tools, GATK (version 2.8) (https://broadinstitute.github.io/picard/)<br>Detection of somatic variants: MuTect (version 1.1.4) (https://software.broadinstitute.org/cancer/cga/mutect_download)<br>Detection of INDELs: VarScan2 (version 2.3.8) (http://varscan.sourceforge.net/)<br>Detections of INDELs: SCALPEL (http://scalpel.sourceforge.net/index.html)<br>Annotation of variants: ANNOVAR (https://annovar.openbioinformatics.org/en/latest/user-guide/download/)<br>Tumour purity, ploidy and copy number estimation: Sequenza (https://cran.r-project.org/web/packages/sequenza/vignettes/sequenza.html)<br>Identification of exonic homopolymers: SciRoKo (version 3.4) (https://kofler.or.at/bioinformatics/SciRoKo/Download.html)<br>HLA haplotyping and mutation calling: Polysolver (Version 4) (https://software.broadinstitute.org/cancer/cga/polysolver)
Data analysis	Tumour phylogenetic reconstruction: Paup (https://paup.phylosolutions.com/)<br>Generation of phylogenetic trees: Figtree (version 1.4.4) (https://github.com/rambaut/figtree/releases)<br>Neoantigen calling: Neopredpipe (https://github.com/MathOnco/NeoPredPipe)<br>Hompolymer read length distribution analysis: MSIsensor (version 0.6) (https://github.com/ding-lab/msisensor/blob/master/README_msisensor.md)<br>Mutation signature analysis: SigProfiler (Version 3.1) (https://github.com/AlexandrovLab)<br>Immune dN/dS analysis: SOPRANO (https://github.com/luisgls/SOPRANO)<br>Subclonal deconvolution and mutation rate analysis: MOBSTER (https://github.com/caravagnalab/mobster)

For manuscripts utilizing custom algorithms or software that are central to the research but not yet described in published literature, software must be made available to editors and reviewers. We strongly encourage code deposition in a community repository (e.g. GitHub). See the Nature Portfolio guidelines for submitting code & software for further information.

Data

Policy information about availability of data

All manuscripts must include a data availability statement. This statement should provide the following information, where applicable:

- Accession codes, unique identifiers, or web links for publicly available datasets
- A description of any restrictions on data availability
- For clinical datasets or third party data, please ensure that the statement adheres to our policy

1. Whole genome sequencing data from the Genomics England colorectal cancer dataset can be accessed by application through the Genomics England Clinical Interpretation Partnership (https://www.genomicsengland.co.uk/about-gecip/joining-researchcommunity/).
2. Variant calls from the TCGA whole exome sequencing dataset can be retrieved from the Genomics Data Commons (GDC) website (https://portal.gdc.cancer.gov/).
3. Whole exome sequencing data from the UCL colorectal cancer cohort has been deposited to the European Genome Phenome archive under accession code:

Research involving human participants, their data, or biological material

Policy information about studies with human participants or human data. See also policy information about sex, gender (identity/presentation), and sexual orientation and race, ethnicity and racism.

Reporting on sex and gender	Reported data in line with UK HTA data and tissue privacy laws
Reporting on race, ethnicity, or other socially relevant groupings	As above
Population characteristics	Populations characteristics in this study:<br>Mismatch repair deficient colorectal cancer<br>Adult (age >18)
Recruitment	UCL WXS cohort: Participants were recruited from the UCL/UCLH biobank of health and disease archive according to Institutional Review Board approved protocol (Project Reference Number NC21.18). No bias to report.<br><br>Genomics England WGS cohort: The participants were recruited across 13 NHS Genomic Medicine Centres and written informed consent was obtained from the participants. No bias to report.
Ethics oversight	Approval provided by UCL/UCLH Biobank of health and disease ethics review committee (Project Reference Number NC21.18)

Note that full information on the approval of the study protocol must also be provided in the manuscript.

Field-specific reporting

Please select the one below that is the best fit for your research. If you are not sure, read the appropriate sections before making your selection.

☒ Life sciences ☐ Behavioural & social sciences ☐ Ecological, evolutionary & environmental sciences

For a reference copy of the document with all sections, see nature.com/documents/nr-reporting-summary-flat.pdf

Life sciences study design

All studies must disclose on these points even when the disclosure is negative.

Sample size	In the Genomics England WGS cohort, we analysed all available colorectal tumours identified as microsatellite instable (MSI) resulting in a total of n=217 tumours.<br><br>In the TCGA cohort, we analysed microsatellite instable (MSI) tumours in the colorectal, uterine, stomach and esophageal datasets resulting in a total of n=181 tumours.<br><br>In the UCL WXS cohort, we identified all mismatch repair deficient (MMRd) colorectal tumours diagnosed between 2014 to 2018 resulting in a total of n=88 tumours with loss of at least one MMR protein. After assessing for tissue quality and tumour block availability, we identified n=11/40 (28%) tumours with subclonal loss of MSH6 on a background of clonal MLH1/PMS2 loss. A stage and age matched cohort of n=11 MH1/PMS2 deficient tumours without evidence of MSH6 loss was used as the control group. Due to the limited sample availability no sample size calculation was performed upfront.
Data exclusions	In the Genomics England cohort we excluded n=6 tumours with discernible pathogenic POLE or POLD1 mutations.<br>In the TCGA cohort we excluded n=9 tumours with discernible pathogenic POLE or POLD1 mutations.
Replication	For analyses of human tumour datasets, replication is not relevant as these specimens cannot be replicated.

Replication	For the patient derived organoid (PDO) analysis a minimum of four independent pairs of parent and daughter PDO lineages were assessed for each of the four MMR genotypes.
Randomization	There was no therapy or intervention in this study, therefore randomisation is not relevant.
Blinding	There was no therapy or intervention in this study, therefore randomisation is not relevant.

Reporting for specific materials, systems and methods

We require information from authors about some types of materials, experimental systems and methods used in many studies. Here, indicate whether each material, system or method listed is relevant to your study. If you are not sure if a list item applies to your research, read the appropriate section before selecting a response.

Materials & experimental systems

n/a	Involved in the study
☐	☒ Antibodies
☐	☒ Eukaryotic cell lines
☒	☐ Palaeontology and archaeology
☒	☐ Animals and other organisms
☒	☐ Clinical data
☒	☐ Dual use research of concern
☒	☐ Plants

Methods

n/a	Involved in the study
☒	☐ ChIP-seq
☒	☐ Flow cytometry
☒	☐ MRI-based neuroimaging

Antibodies

Antibodies used	MSH6: Clone: EP49, Company: Agilent, Catalog: M364601-2<br>MLH1: Clone: ES05, Company: Agilent, Catalog number: M364001-2<br>PMS2 Clone: A16-4 Company: BD Sciences, Catalog number: 556415<br>MSH2 Clone: FE11 Company: Agilent, Catalog number: M363929-2<br>MSH3 Clone: 611390 Company: BD Sciences, Catalog number: 611390<br>CD20: Clone:L26, Company: Agilent, Catalog number: M0755<br>FOXP3: Clone: D608R, Company: Cell Signalling Technology, Catalog number: 12653<br>CD4: Clone: 4B12, Company: Agilent, Catalog number: M7310<br>PANCK: Clone: AE1/3, Company: Agilent, Catalog number: M3515<br>CD8: Clone: 4B11, Company: Agilent, Catalog number: M7103
Validation	All antibodies were validated using positive and negative control tissue. For the MMR proteins MLH1, PMS2, MSH2 and MSH6, these have been extensively validated by HSL-AD which runs the clinical diagnostic laboratory at our centre and we used the same antibody clones and IHC conditions as validated for clinical samples. For immune cell markers CD8, CD4, FOXP3 and CD20, these were validated using lymph nodes tissue and normal colonic mucosa.

Eukaryotic cell lines

Policy information about cell lines and Sex and Gender in Research

Cell line source(s)	Patient derived (identity not disclosed)
Authentication	N/A
Mycoplasma contamination	Routinely tested
Commonly misidentified lines<br>(See ICLAC register)	N/A

Plants

Seed stocks	*Report on the source of all seed stocks or other plant material used. If applicable, state the seed stock centre and catalogue number. If plant specimens were collected from the field, describe the collection location, date and sampling procedures.*
Novel plant genotypes	*Describe the methods by which all novel plant genotypes were produced. This includes those generated by transgenic approaches, gene editing, chemical/radiation-based mutagenesis and hybridization. For transgenic lines, describe the transformation method, the number of independent lines analyzed and the generation upon which experiments were performed. For gene-edited lines, describe the editor used, the endogenous sequence targeted for editing, the targeting guide RNA sequence (if applicable) and how the editor was applied.*
Authentication	*Describe any authentication procedures for each seed stock used or novel genotype generated. Describe any experiments used to assess the effect of a mutation and, where applicable, how potential secondary effects (e.g. second site T-DNA insertions, mosiacism, off-target gene editing) were examined.*
